# Social approach and place aversion in relation to conspecific pain in dairy calves

**DOI:** 10.1371/journal.pone.0232897

**Published:** 2020-05-14

**Authors:** Thomas Ede, Marina A. G. von Keyserlingk, Daniel M. Weary

**Affiliations:** Animal Welfare Program, Faculty of Land and Food Systems, University of British Columbia, Vancouver, B.C., Canada; University of Illinois, UNITED STATES

## Abstract

Despite scientific interest in animal empathy, and growing public concern for farm animal welfare, the empathic abilities of farm animals remain under researched. In this study, we investigated empathic responses of young Holstein dairy calves to conspecifics recovering from hot-iron disbudding, a painful procedure common on dairy farms. A combination of social approach and place conditioning was used. First, ‘observer’ calves witnessed two ‘demonstrator’ calves recover from either a painful procedure (hot-iron disbudding and sedation) or a sham procedure (sedation alone) in distinct pens. Observer calves spent more time in proximity and paid more attention to calves recovering from the painful procedure compared to sham calves (proximity: 59.6 ± 4.3%; attention: 54.3 ± 1.5%). Observers were then tested for conditioned place aversion (in the absence of demonstrators) at 48h, 72h and 96h after the second demonstration; observers tended to avoid the pen associated with conspecific pain during the second of the three tests, spending 34.8 ± 9.6% of their time in this pen. No strong evidence of pain empathy was found, but our tentative results encourage further research on empathy in animals.

## Introduction

Assessing animal empathy is not straightforward, in part because the definition of empathy is subject to disagreement [1–3]. In this study, we consider empathy in its broadest sense: a multi-layered sensitivity to a conspecific’s state, ranging from basic mimicry to more complex perspective-taking (i.e. ‘imagining yourself in the physical or mental place of another’) [4–6]. Many approaches have been adopted in the study of empathy, from consolation in primates after conflict [7–9], to rats freeing mates from traps [10–12]. Farm animals have rarely been studied [5,6], and to our knowledge no work on cattle has been published. Most farm animals are gregarious, including cattle, so the social environment is likely to be relevant [13]. Moreover, cattle are routinely subjected to painful procedures, including hot-iron disbudding for dairy calves [14,15], providing an opportune model to explore empathic responses to pain.

This study had three aims. First, to investigate whether calves preferentially associate with a conspecific in pain compared to an unaffected conspecific (*Objective 1*). Based on previous observations in mice [16,17], we predicted calves would preferentially approach a conspecific in pain. Second, in an effort to examine a more complex empathic process (defined as ‘true empathy’ by Edgar et al. [18]), we tested whether the empathic response was valenced (i.e. positive or negative) by using conspecific state as a conditioning stimuli in a place conditioning paradigm [19] (*Objective 2*). As previously shown in mice, we predicted that a conspecific state of pain would result in conditioned place aversion [17]. Finally, we examined whether calf empathic responses are dependent on ‘pain-related’ behaviours [15] commonly used in the assessment of calf pain following disbudding. We predicted that calves showing more pain behaviours would be more attractive as a social partner and elicit stronger conditioned aversion.

## Methods

### Ethics statement

This research was conducted at the University of British Columbia’s Dairy Education and Research Center in Agassiz, Canada. All procedures were approved by the university’s Animal Care Committee (under protocol A16-0310).

### Animals and housing

Female Holstein calves were housed in group pens (4.9 x 7.3 m) of 8 to 10 animals. Calves had been living in the same pen since they were 7 days old. Calves (n = 36) were enrolled as trios (n = 12) coming from the same pen: one ‘observer’ (n = 12) and two ‘demonstrators’ (n = 24). The average age and weight at enrollment were 47 (± 5.0) days and 75.0 (± 10.2) kg, with an age difference within the trio of 3 (± 2.8) days.

### Apparatus

**Fig 1:**
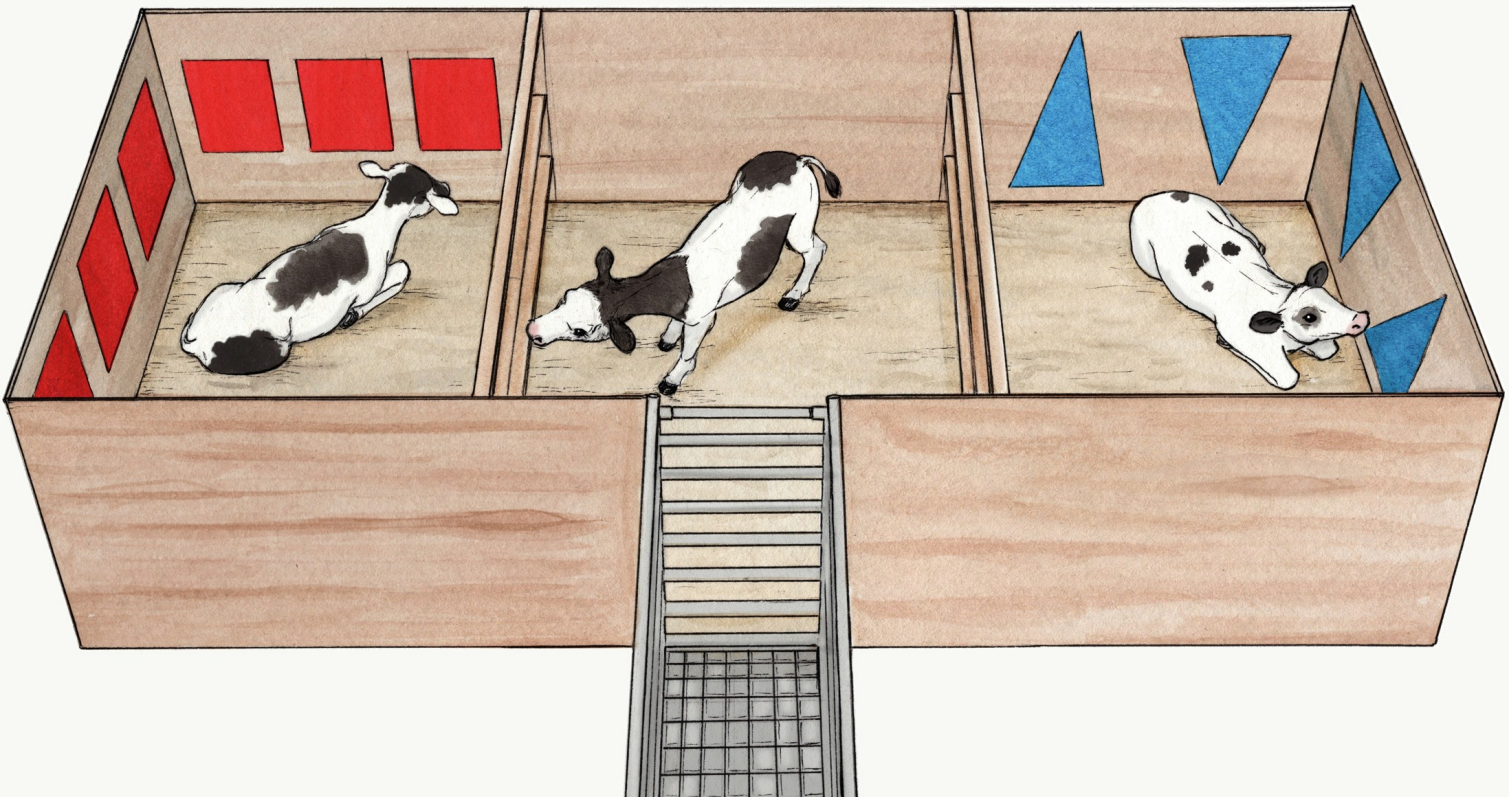
Experimental apparatus. During demonstrations, observer calves were restricted in the central pen and demonstrators were restricted in treatment pens (with either red squares or blues triangles on the walls). One demonstrator was recovering from a painful procedure (sedation, local anesthesia and hot-iron disbudding) and the other from a sham procedure (sedation alone). During test sessions gates were removed, and observers were assessed by themselves for conditioned place aversion. Illustration by Ann Sanderson.

The apparatus was a 2.1 x 6.0 m area divided in three 2.1 x 2.0 m pens connected by removable gates that calves could see and interact through (horizontal fences, made of three 38 x 89 mm wood studs). The outermost sections were treatment pens, with distinct visual cues on the walls (either two blue triangles or three red squares) to help calves make the association between pen and treatment. Calves entered the apparatus through the central pen accessed via a chute acting as a start-box (Fig 1).

### Protocol

#### Pre-exposure

Observer calves were individually pre-exposed to the apparatus. Observers were led from their home pen to the start-box where they received a small (0.3 L) milk reward. They were then let into the apparatus, with gates removed, allowing free access to all three sections. Time spent in each section (with both front legs in the pen) was recorded throughout the 15 min trial, and calves were then returned to their home pen. To reduce the risk of pre-existing avoidance bias, two calves that did not enter all pens during pre-exposure were not enrolled.

#### Demonstrations

Each trio (one observer and two demonstrators) were subjected to two demonstrations 24 h and 72 h after observer pre-exposure. During demonstrations, gates separating the pens were in place such that the observer calf was restricted to the central pen and demonstrators were each confined to one of the two treatment pens (as illustrated in Fig 1). Over the course of the 6 h trial, the observer could see and have limited physical contact (i.e. head contact through gates) with demonstrators recovering from two different treatments: ‘sham’ calves that had been sedated with xylazine (0.2 mg/kg BW, Rompun, 20 mg/mL, Bayer, Leverkusen, Germany), and ‘pain’ calves that had received the same sedation, as well as local anesthesia (5 mL of Lido-2; lidocaine 2%, Epinephrine 1:100,000, Rafter8, Calgary, Canada; injected in the lateral canthus of each eye) and hot-iron disbudding (X30 Rhinehart, Spencerville, IN, USA; heated to approximately 500°C and applied to horn buds for approximately 15 s, 10 min after local anesthesia). During the second demonstration (which also lasted 6 h), the observer was again restricted to the central pen but demonstrators were placed in the opposite pen and were assigned the opposite treatment (i.e. a demonstrator that first received the ‘pain’ treatment would next receive the ‘sham’ treatment and vice-versa). This design was chosen to balance the social preferences of observers for specific demonstrators. Colour of the treatment pen associated with the ‘pain’ procedure was balanced across trios (n = 6 in red squares, n = 6 in blue triangles). Preferences observed during pre-exposure were also balanced across treatments.

All observers had previously been disbudded (following the same ‘pain’ procedure that demonstrators experienced) two days before enrollment.

#### Tests

Observers were tested for conditioned place aversion 48h, 72h and 96h after the second demonstration. Gates were removed so observers could freely explore the apparatus until they chose to lie down for at least one minute, or 60 min had passed (which ever occurred first); calves were then returned to their home pen.

### Measures and statistical analysis

All calves were video recorded during demonstration and test trials (camera: WV-CP310, Panasonic Canada, Ontario). Videos were analysed using Geovision’s viewlog software (Vision Systems, Saint-Laurent, Canada) by one blinded and one non-blinded observer. Proximity, attention and contact of the observer with the demonstrators were recorded. Pain behaviours displayed by demonstrators were also recorded (see Table 1 for details). Inter-rater agreements were calculated for proximity, attention, interaction and pain behaviours based on over 100 observations, using R’s ‘agree’ function [20]. During tests, time spent in each pen by the observer was continuously recorded, as well as where observers chose to lie down.

**Table 1:** Behaviours recorded during demonstrations (where an ‘observer’ calf would witness two ‘demonstrators’ recover from either a painful or a sham procedure) and during tests (where observers were tested for conditioned place aversion of the pens associated with the two procedures).

Measure	Session	Subject calf	Description	Sampling method
Proximity	Demonstration	Observer	Which half of the central pen the calf placed her front legs	Instantaneous scans every 5 min
Attention	Demonstration	Observer	Which demonstrator the calf’s head was oriented towards	Instantaneous scans every 5 min
Contact	Demonstration	Demonstrator	Number of physical contacts between observer and demonstrator	1 min scans every 5 min
Pain behaviours	Demonstration	Demonstrator	Number of ear flicks, head rubs and head shakes	1 min scans every 5 min
Conditioned place aversion	Test	Observer	Time spent in each pen, where the calf lay down	Continuous

Preferences in proximity, attention and contact for the conspecific recovering from disbudding were calculated for each observer as a ratio of the number of scans directed towards the ‘pain’ demonstrator compared to the total number of scans directed towards both demonstrators (i.e. the sum of scans directed towards ‘pain’ demonstrators and ‘sham’ demonstrators). A difference from the null expectation of 50% preference was calculated with a one-sample Student t-test. Similarly, during conditioned place aversion tests preference for the pen associated with conspecific pain was calculated as a ratio of time spent in the ‘pain’ pen compared to the total amount of time spent in both treatment pens. A difference from the null expectation of 50% preference was again tested with a one-sample t-test. Differences in where calves chose to lie down were analysed with Pearson χ² tests.

Due to low counts, pain behaviours were summed to calculate the total numbers of pain behaviours displayed in the ‘pain’ and ‘sham’ pen for each trio. Pain behaviours were analysed with a t-test to confirm that these behaviors differed with treatment. To test if observer preferences (during demonstrations and place aversion tests) were associated with these behaviors, we calculated the difference in the pain behaviours witnessed by the observer (i.e. the total in the pain pen minus the total is the sham pen, across the two demonstrator sessions) and compared this with preference using Pearson correlation. Data were graphically scrutinized for normality and outliers.

## Results

Inter-rater agreement was satisfactory for all measures (position: 96%, attention: 83%, interaction: 100%, pain behaviours: 96%).

### *Objective 1*: Do calves preferentially associate with a conspecific in pain?

**Fig 2:**
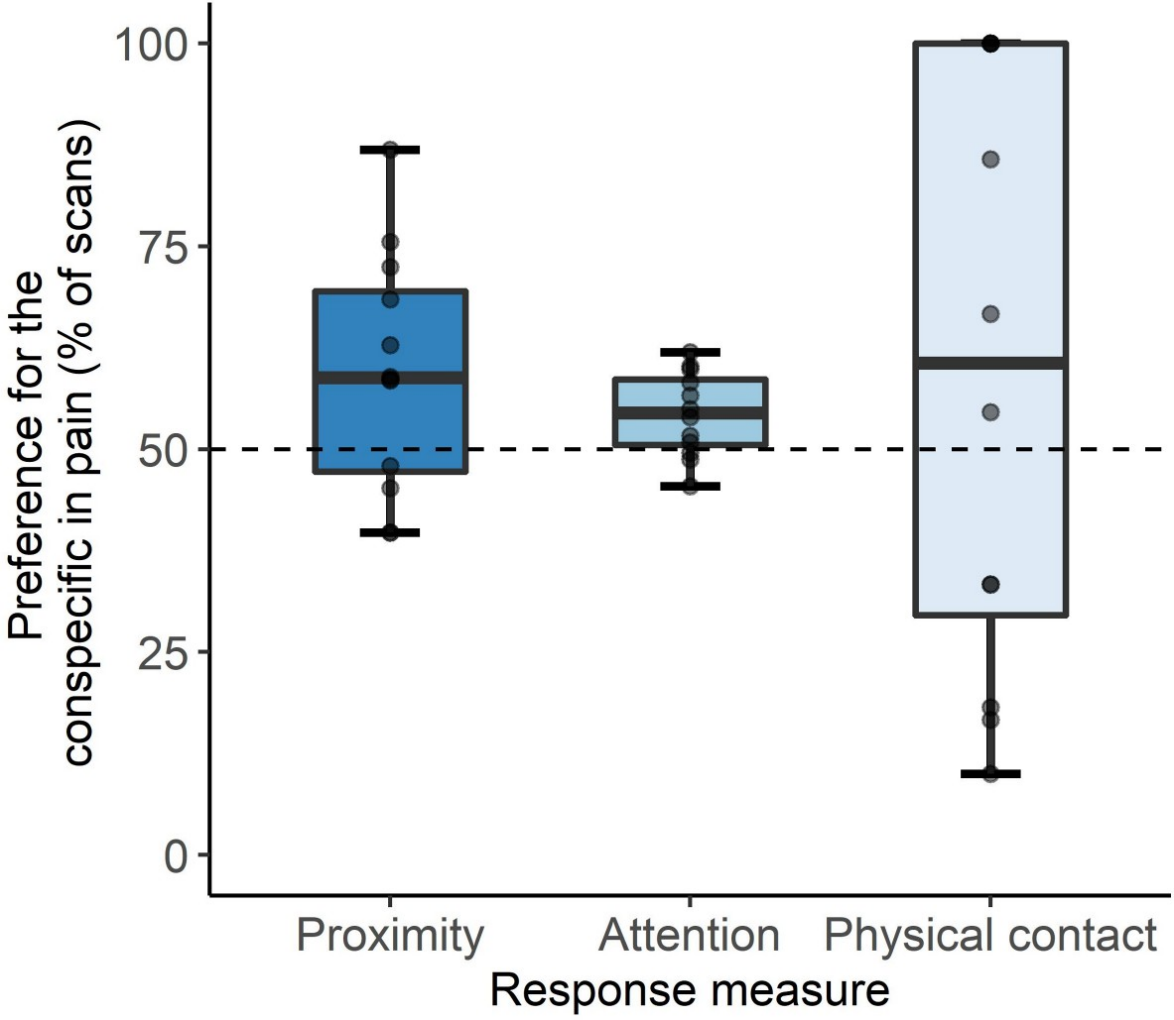
Preferences of proximity, attention and contact of observer calves towards conspecifics in pain (recovering from sedation, local anesthesia and hot-iron disbudding) compared to sham conspecifics (recovering from sedation only). Values above 50% represent a preference for the conspecific in pain.

During demonstrations, observers spent more time in proximity and paid more attention to conspecifics recovering from disbudding compared to what could be expected by chance (Fig 2; mean ± SE proximity: 59.6 ± 4.3% of scans, t_11_ = 2.2, P = 0.05; attention: 54.3 ± 1.5% of scans, t_11_ = 2.9, P = 0.01). Physical contact occurred infrequently (on average just 5.2 ± 1.1 contacts per trio); 59.9 ± 10.5% of these contacts were with the painful calf (t_11_ = 0.9, P = 0.4).

### *Objective 2*: Does observing a conspecific in pain lead to conditioned place aversion?

**Fig 3:**
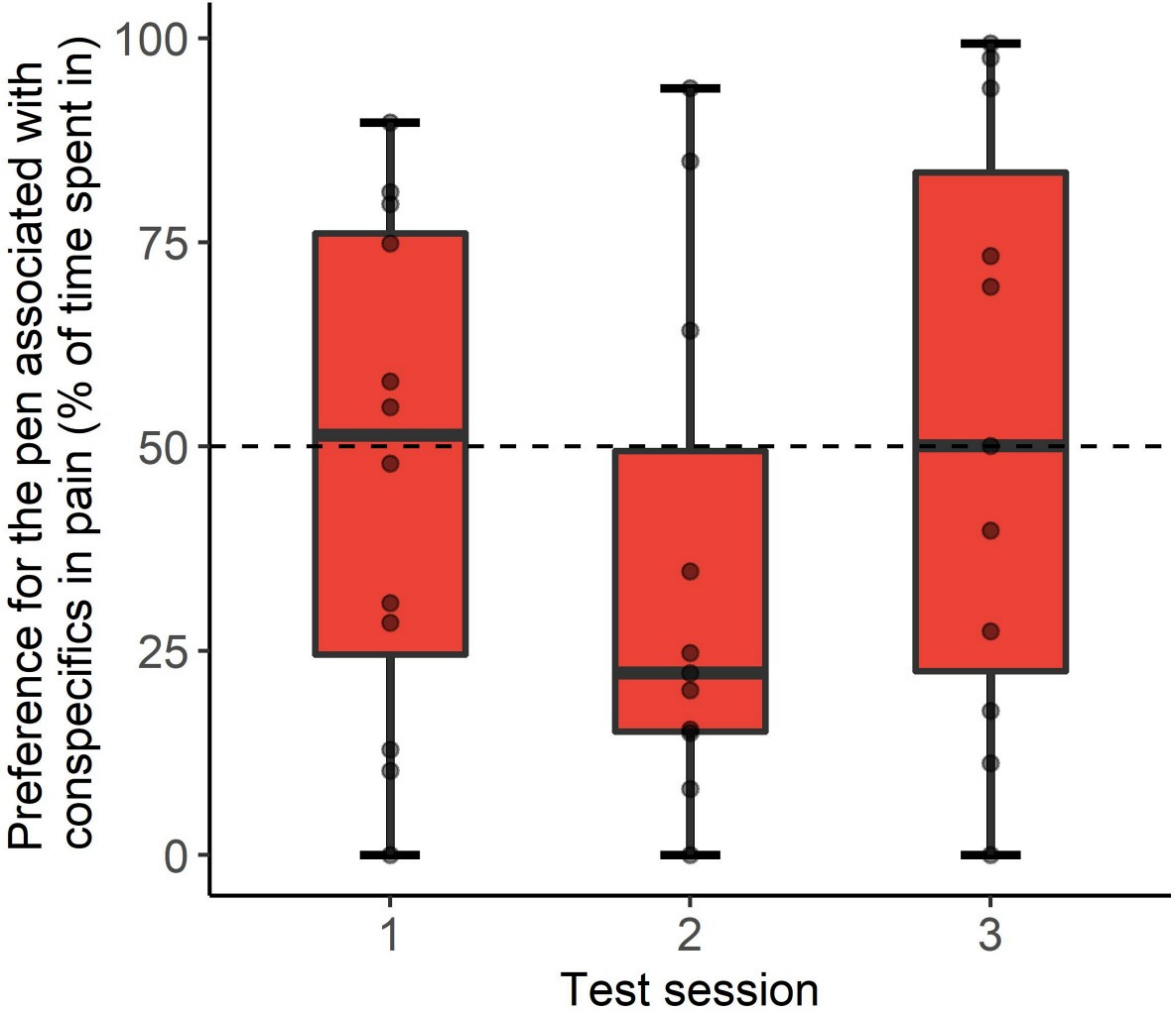
Conditioned place aversion results. Preference in time spent by observer calves in the pen where they previously observed pen mates recover from the ‘pain’ procedure (sedation, local anesthesia and hot-iron disbudding) compared to the ‘sham’ procedure (sedation alone). Values under 50% represent an aversion to the ‘pain’ pen. Test sessions 1, 2 and 3 took place 48 h, 72 h and 96 h after the last demonstration.

Observers tended to spend less time in the pen associated with the conspecific in pain compared to what could be expected by chance, but only during the second aversion test (Fig 3; 34.8 ± 9.6% of time spent in treatment pens, t_10_ = -1.6, P = 0.1) with no difference detected during the first and third tests (first test: 47.4 ± 8.8%, t_11_ = -0.3, P = 0.8; third test: 52.7 ± 10.9%, t_10_ = 0.2, P = 0.8).

Out of the 36 tests (12 calves x 3 sessions), calves did not lie down within the 60 min period provided on 10 occasions, and lay down in the central pen in six of the sessions. Out of the remaining 20 sessions, calves lay down 11 times in the ‘sham’ pen and 9 times in the ‘pain’ pen, χ² = 0.2, P = 0.7). In all cases calves lay down for at least one minute, ending the session.

### Objective 3: Does observer response vary in relation to the demonstrators’ pain-related behaviour?

**Fig 4:**
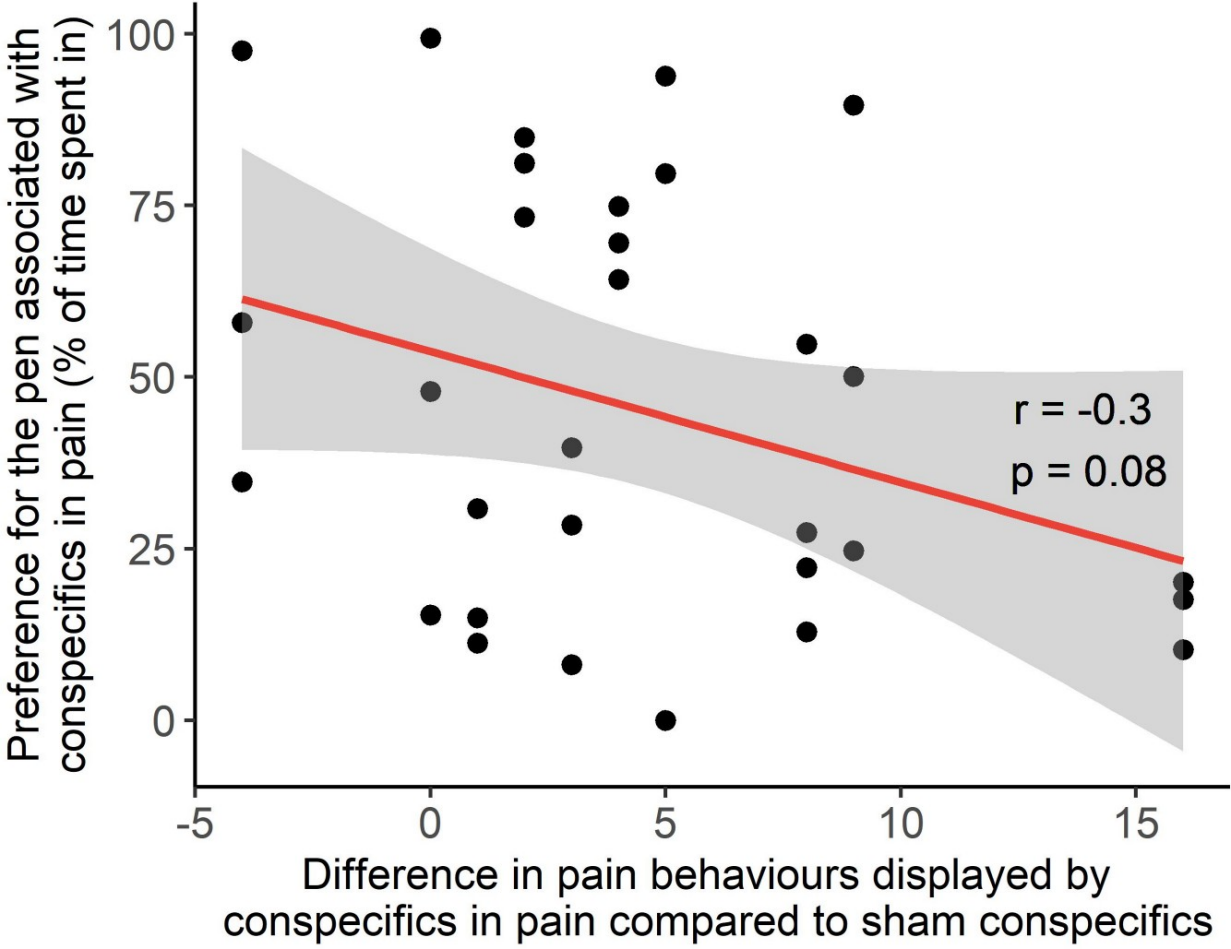
Correlation between the difference of pain behaviours displayed by demonstrators in the ‘pain’ procedure (sedation, local anesthesia and hot-iron disbudding) and ‘sham’ (sedation alone) procedures, versus observer calf place aversion of the pen associated with pain. The shaded area represents the 95% confidence interval of the regression.

Demonstrators recovering from disbudding displayed more pain behaviours compared to calves recovering from the ‘sham’ procedure (4.8 ± 1.5 more pain behaviours; t_11_ = 3.2, P = 0.008). The difference in pain behaviours displayed by ‘pain’ demonstrators compared to ‘sham’ demonstrators was not correlated with preference in position, attention or contact of the observer (position: r = -0.2, P = 0.6; attention: r = 0.03, P = 0.9; contact: r = -0.09, P = 0.8). During place aversion tests, observers tended to show stronger avoidance of the pen associated with pain if more pain behaviours were displayed (Fig 4; r = -0.3, P = 0.09).

## Discussion

### *Objective 1*: Do calves preferentially associate with a conspecific in pain?

Our results indicate that dairy calves are socially attracted to animals in pain, as evidenced by the increased time spent in proximity and attention to demonstrators recovering from disbudding, compared to demonstrators recovering from sedation alone. These results are consistent with those of Langford et al. [16] and Watanabe et al. [17] who reported that mice are more likely to approach cage-mates in pain.

### *Objective 2*: Does observing a conspecific in pain lead to conditioned place aversion?

Place aversion results were mixed, making it difficult to draw strong conclusions. We found no difference in time spent between treatment pens in the first session. We expected aversion to the pain pen to be strongest during the first test, based upon our previous observations of calf avoidance of a pen associated with their own disbudding [21]. However, in the current study observers were restricted to the central pen during demonstrations, so calves may have been motivated to explore the unfamiliar test pens when provided the opportunity in the first test session, allowing for treatment effects to emerge in the second session when calves were less motivated to explore. We encourage future studies to provide calves with habituation sessions to the test apparatus before the observer sessions to diminish any effects of novelty.

The finding from the second session (i.e. that calves tended to avoid the pen associated with conspecific pain), warrants further study on the idea that the affective state of another calf can act as a conditioning stimulus. During the third and last test, the lack of place aversion was predicted and is consistent with previous work on conditioned pain preference in calves [21], as pens were expected to lose their association to treatment with repeated, unreinforced test sessions.

### Objective 3: Does observer response vary in relation to the demonstrators’ pain-related behaviour?

Demonstrators recovering from disbudding displayed more pain-related behaviours than sham demonstrators, a result that is consistent with the considerable research on hot-iron disbudding of calves [14]. We found no evidence that the expression of these behaviors was associated with observer proximity, attention or contact with the demonstrators, but calves that observed demonstrators showing stronger pain responses tended to show stronger place aversion to the pen associated with disbudding.

Previous reports of animal empathic processes are ambiguous: increased contact of ewes with a lamb in pain was correlated with the lamb’s pain behaviours [22], but ‘fear conditioning by proxy’ of rats (i.e. learning to fear a tone by being exposed to demonstrators who had previously associated the tone with an electric shock) was not dependent on the number of fear behaviours displayed by demonstrators [23]. Similarly, observer mice did not require visual cues to develop hyperalgesia when placed in a room with another mouse in pain [24]. Langford et al. [16] reported a negative correlation between the proximity of observers and the number of pain behaviours expressed by demonstrators. The type of cues relevant to different species likely varies among species (e.g. rodents may rely less on visual cues as they tend to have a poorer eyesight). Our results suggest that calves are able to identify a conspecific in pain but may do so using cues other than the pain behaviors we measured in the current study.

### General discussion

Emotional contagion is a process by which observers experience similar states to those experienced by demonstrators, as illustrated, for example, by mice becoming hyperalgesic in the presence of other mice in pain [24]; this is considered a basic form of empathy [5,6]. In the case of emotional contagion, we would not expect observers to preferentially approach demonstrators in pain as they are the source of their own discomfort. As we observed approach towards the conspecific in pain, our results indicate that the empathic response of calves is not limited to basic motor mimicry or emotional contagion (or ‘primal empathy’ [1]) but rather includes a component of perspective taking, implying the capacity to separate the conspecific’s situation from their own. The tendency of observers to also avoid a place associated with conspecific pain suggests that calves might be able to identify that a conspecific is in a negative state and use this information to learn about their surroundings.

There are many limitations to the current study. We used a relatively low sample size for both ethical (calves were not provided with drugs to control post-operative pain) and practical reasons. Moreover, the experimental design restricted social contact between observers and demonstrators to only head contact. Calves are motivated for full social contact [25], meaning that the restriction imposed in this study might have impaired calves empathic process. This restriction might also explain the low number of physical contacts observed. It is also unclear what, if any, effect the smell of disbudding may have had on the observer’s response. Finally, the removal of demonstrators during tests may have affected the conditioned aversion response of observers.

Additional factors should be considered in future work. We explored the relationship between young, unrelated animals but it is reasonable to predict a greater empathic response from animals with better established bonds, such as a dam towards her offspring [26]; other work has reported increased empathic responses with increased kinship and familiarity [8,16,27,28]. A sex effect has been observed in mice, with males showing less evidence of empathy [16]. Only female calves were enrolled in the current study preventing any inferences regarding sex. Finally, observer calves had all been previously disbudded, potentially affecting their response; previous experience of pain and distress has been indicated to influence empathic response in other species [11,29].

The focus of this study was the effect of the conspecific’s state on the observer, but it would be interesting to explore whether the observers’ response (or simply social presence) affects demonstrators in their recovery from a painful procedure. We speculate that social presence will facilitate recovery from pain; evidence of ‘social buffering’ has previously been described in humans, primates, rodents and birds [30], including effects specific to pain mitigation in humans, rats, mice and goats [31].

## Conclusion

Calves spent more time in proximity and paid more attention to a conspecific in pain compared to a sham treated calf, and tended to avoid the pen associated with conspecific pain, especially when more ‘pain-related’ behaviours were shown by the calf. These results are suggestive of an empathic processes that warrants further study.

## Supporting information

S1 Data (CSV)

S2 Data (CSV)

S3 Data (DOCX)

S4 Data (R)
