# Supplementary material for: Social approach and place aversion in relation to conspecific pain in dairy calves
Source: PLoS One. 2020 May 14;15(5):e0232897. doi: 10.1371/journal.pone.0232897 (PMC7224486; doi:10.1371/journal.pone.0232897)
Supplement: S3 Data — (DOCX) [file pone.0232897.s003.docx]

‘Position attention interaction’ dataset

- Observer_ID: Identification number of the observer calf
- Attention_pain: proportion of scans the observer spent paying attention (i.e. looking toward) to the conspecific in pain (compared to the total number of scans the observer spent paying attention to either demonstrators)
- Position_pain: proportion of scans the observer spent in proximity to the conspecific in pain (compared to the total number of scans the observer spent in proximity to either demonstrators)
- Interaction_pain: proportion of scans the observer spent interacting with the conspecific in pain (compared to the total number of scans the observer spent interacting with either demonstrators)
- Difference_pain_behaviours: difference of pain behaviours (ear flicks, head rubs, head shakes) displayed by the demonstrator in pain compared to the sham calf

‘Place aversion tests’ dataset

- Observer_ID: Identification number of the observer calf
- Session: Conditioned place aversion test number (1,2 and 3. 24h, 72h and 96h after the last conditioning session)
- Time_pain: proportion of time spent in the pen associated with conspecific pain (compared to the total time spent in either treatment pens)
- Difference_pain_behaviours: difference of pain behaviours (ear flicks, head rubs, head shakes) displayed by the demonstrator in pain compared to the sham calf
